# Oral Delivery of a Synthetic Sterol Reduces Axonopathy and Inflammation in a Rodent Model of Glaucoma

**DOI:** 10.3389/fnins.2017.00045

**Published:** 2017-02-07

**Authors:** Wendi S. Lambert, Brian J. Carlson, Cathryn R. Formichella, Rebecca M. Sappington, Clarence Ahlem, David J. Calkins

**Affiliations:** ^1^Vanderbilt University Medical Center, The Vanderbilt Eye InstituteNashville, TN, USA; ^2^NeurMedix, Inc.San Diego, CA, USA

**Keywords:** glaucoma, neuroinflammation, neuroprotection, axonopathy, HE3286, NF kappa B, axonal transport, brain derived neurotrophic factor

## Abstract

Glaucoma is a group of optic neuropathies associated with aging and sensitivity to intraocular pressure (IOP). The disease is the leading cause of irreversible blindness worldwide. Early progression in glaucoma involves dysfunction of retinal ganglion cell (RGC) axons, which comprise the optic nerve. Deficits in anterograde transport along RGC axons to central visual structures precede outright degeneration, and preventing these deficits is efficacious at abating subsequent progression. HE3286 is a synthetic sterol derivative that has shown therapeutic promise in models of inflammatory disease and neurodegenerative disease. We examined the efficacy of HE3286 oral delivery in preventing loss of anterograde transport in an inducible model of glaucoma (microbead occlusion). Adult rats received HE3286 (20 or 100 mg/kg) or vehicle daily via oral gavage for 4 weeks. Microbead occlusion elevated IOP ~30% in all treatment groups, and elevation was not affected by HE3286 treatment. In the vehicle group, elevated IOP reduced anterograde axonal transport to the superior colliculus, the most distal site in the optic projection, by 43% (*p* = 0.003); HE3286 (100 mg/kg) prevented this reduction (*p* = 0.025). HE3286 increased brain-derived neurotrophic factor (BDNF) in the optic nerve head and retina, while decreasing inflammatory and pathogenic proteins associated with elevated IOP compared to vehicle treatment. Treatment with HE3286 also increased nuclear localization of the transcription factor NFκB in collicular and retinal neurons, but decreased NFκB in glial nuclei in the optic nerve head. Thus, HE3286 may have a neuroprotective influence in glaucoma, as well as other chronic neurodegenerations.

## Introduction

Glaucoma is the leading cause of irreversible blindness worldwide (Quigley and Broman, 2006). The disease selectively targets retinal ganglion cells (RGCs) and their axons through stress conveyed at the optic nerve head (Calkins, 2012; Nickells et al., 2012). Age is a major risk factor for developing glaucoma, but sensitivity to intraocular pressure (IOP) is the only modifiable risk factor and sole target for clinical intervention (Heijl et al., 2002). Decreasing IOP with drugs or surgery can slow disease progression, but for many patients does not prevent RGC degeneration and vision loss (Heijl et al., 2002). Given that by 2020 nearly 80 million people will have glaucoma, with an estimated 11.2 million with permanent vision loss (Quigley and Broman, 2006; Cheung et al., 2008; Schober et al., 2008), the identification of therapeutic agents that can protect RGCs independently of IOP is of paramount importance.

HE3286 (17α-Ethynyl-androst-5ene-3β, 7β, 17β-triol) is a synthetic derivative of androst-5-ene-3β,7β,17β-triol (AET), a non-glucocorticoid anti-inflammatory metabolite of the adrenal steroid, dehydroepiandrosterone (DHEA; Ahlem et al., 2011). DHEA and AET have shown therapeutic promise in rodent models of immune-mediated inflammatory disorders with varying results in clinical trials (Offner et al., 2002; Dillon, 2005; Ahlem et al., 2009). HE3286 shows better oral bioavailability in humans, has low potential for toxicity, does not bind to glucocorticoid receptor or any known nuclear hormone receptor, and is not immunosuppressive (Wang et al., 2010; Ahlem et al., 2011). Treatment with HE3286 improves outcome measures in models of autoimmune disease, lung inflammation, experimental optic neuritis and Parkinson's disease, and is currently being tested in clinical trials (Auci et al., 2007, 2010; Ahlem et al., 2009; Offner et al., 2009; Conrad et al., 2010; Lu et al., 2010; Kosiewicz et al., 2011; Nicoletti et al., 2012; Reading et al., 2013a,b; Khan et al., 2014). It is believed HE3286 may act via binding, regulation and/or activation of MAPK or ERK, or through modulation of NFκB (Lu et al., 2010; Ahlem et al., 2011; Nicoletti et al., 2012; Reading et al., 2012).

Neurodegeneration in glaucoma shows many similarities with other age-related neurodegenerative disorders, including early deficits in axon function that precede loss of neurons themselves (Crish and Calkins, 2011; Mckinnon, 2012; Ghiso et al., 2013; Jindal, 2013; Danesh-Meyer and Levin, 2015; Jain and Aref, 2015). In animal models of glaucoma, degradation of RGC anterograde transport to central brain targets occurs early and prior to outright loss of axons in the optic projection (Crish et al., 2010, 2013; Dengler-Crish et al., 2014). Experimental interventions that prevent transport deficits are efficacious at stopping subsequent degeneration of axons and cell bodies (Lambert et al., 2011; Dapper et al., 2013). Here, we tested whether HE3286 could rescue RGC axon transport and modulate markers for neuroinflammation in the optic projection using our inducible microbead occlusion model of glaucoma in rats (Crish et al., 2010; Sappington et al., 2010; Dapper et al., 2013). We found that while HE3286 had no effect on IOP, daily oral delivery for 4 weeks prevented deficits in anterograde transport to the superior colliculus (SC), the primary central projection for RGCs in rodents (Linden and Perry, 1983; Hofbauer and Drager, 1985). HE3286 also influenced the level of many proteins implicated in the pathogenesis of glaucoma and other neurodegenerative disorders throughout the optic projection. Finally, HE3286 increased activation of the transcription factor NFκB in colliculus and retina, while decreasing glial NFκB activation in the optic nerve head, which is a major pathogenic site of neuroinflammation in glaucoma (Soto and Howell, 2014; Russo et al., 2016). These data suggest HE3286 has therapeutic potential with regards to neurodegeneration in glaucoma by modulating common neuroinflammatory pathways.

## Materials and methods

### Animals

All experimental procedures were conducted in accordance with the guidelines of and approved by The Vanderbilt University Institutional Animal Care and Use Committee. Brown Norway rats (7–9 months old, male) were obtained from Charles River Laboratories (Wilmington, MA) and maintained in a 12-h light-dark cycle with standard rodent chow available *ad libitum*. Three cohorts of rats (*n* = 6 per cohort; 18 rats total) were randomly assigned to one of three treatment groups: vehicle, 20 mg/kg HE3286 or 100 mg/kg HE3286. We measured IOP bilaterally in awake rats using a TonoPen XL rebound tonometer (Medtronic Solan, Jacksonville, FL) as previously described (Sappington et al., 2010; Crish et al., 2013; Dapper et al., 2013). To avoid corneal irritation, hydrating eye drops were administered to each eye at the completion of IOP measurements. Prior to microbead occlusion (Sappington et al., 2010; Crish et al., 2013; Dapper et al., 2013), we monitored IOP for 2–3 days; these measurements were averaged to obtain a baseline value. We elevated IOP unilaterally (OS) by a single 5.0 μl injection of 15 μm polystyrene microbeads (Molecular Probes, Eugene, OR) into the anterior chamber. The fellow eye (OD) received an equivalent volume of saline to serve as an internal control. Beginning 24 h post-injection (day 1), we monitored IOP using tonometry at least three times weekly for the duration of the experiment (Sappington et al., 2010; Crish et al., 2013; Dapper et al., 2013). Beginning with the microbead injection (day 0), rats received 20 mg/kg or 100 mg/kg HE3286 (10 mg/mL HE3286 in an aqueous medium containing 1 mg/mL sodium carboxymethyl cellulose, 9 mg/mL sodium chloride, 20 mg/mL polysorbate-80, and 0.5 mg/mL phenol as abroad spectrum preservative, Harbor Therapeutics, San Diego, CA 92122) via oral gavage. For the vehicle group, half received 20 mg/kg vehicle and the other half 100 mg/kg vehicle (1 mg/mL sodium carboxymethyl cellulose, 9 mg/mL sodium chloride, 20 mg/mL polysorbate-80, and 0.5 mg/mL phenol in an aqueous medium, Harbor Therapeutics, San Diego, CA 92122). Rats received vehicle or HE3286 once daily via oral gavage for 28 days.

### Anterograde axonal transport

Forty-eight hours prior to perfusion, rats were anesthetized with 2.5% isoflurane and injected intravitreally with 2 μl of 0.5 mg cholera toxin subunit B (CTB) conjugated to Alexa Fluor-488 (Molecular Probes, CA) as previously described (Crish et al., 2010; Dapper et al., 2013; Ward et al., 2014). Animals were transcardially perfused with phosphate buffered saline (PBS) followed with 4% paraformaldehyde in PBS. Brains were cryoprotected overnight in 30% sucrose/PBS and coronal midbrain sections (50 μm) cut on a freezing sliding microtome. Alternating sections of superior colliculus (SC) were imaged using a Nikon Ti Eclipse microscope (Nikon Instruments Inc., Melville, NY) and the intensity of CTB signal was quantified using a custom ImagePro macro (Media Cybernetics, Bethesda, MD) as previously described (Crish et al., 2010; Dapper et al., 2013; Ward et al., 2014). After normalizing to background, CTB signal intensity was calculated to reconstruct a retinotopic map of intact anterograde transport across the SC. Percent of intact transport for each map was defined as the region of the SC with intensity ≥70% of the maximum CTB signal for that tissue. CTB uptake by RGCs in the retina was verified using a Zeiss FV-1000 inverted confocal microscope through the Vanderbilt University Medical Center Cell Imaging Shared Resource.

### Immunohistochemistry

Whole eyes were dissected from perfused animals, paraffin-embedded and vertically sectioned (6 μm). Immunohistochemistry of whole eyes and brain was performed as previously described and at identical conditions between cohorts (Sappington et al., 2009; Crish et al., 2010; Weitlauf et al., 2014). Primary antibodies used to immunolabel proteins of interest in the brain, optic nerve head, and retina are listed in Table 1. Sections were incubated with appropriate secondary antibodies (1:200; Jackson ImmunoResearch Laboratories, Inc., West Grove, PA) and then cover-slipped with DAPI Fluoromount G (Southern Biotech, Birmingham, AL). Sections were imaged using a Zeiss FV-1000 inverted confocal microscope through the Vanderbilt University Medical Center Cell Imaging Shared Resource. For retina, all images were collected within the mid-peripheral region for comparison across eyes and cohorts. Identical microscope settings were used to acquire images for signal quantification, which was performed by a naïve observer using a custom macro in ImagePro (Media Cybernetics; Bethesda, MD) that determines the percent area of the positive label (Crish et al., 2013). The microbead to saline ratio (microbead:saline) of label in a specific tissue was calculated for each animal; ratios were averaged and reported as label (microbead:saline). Nuclear localization of NFkB was analyzed using a custom macro in ImageJ (Carmona et al., 2007; Schneider et al., 2012) followed by signal quantification in ImagePro. For quantifying label, a tissue section serving each saline and microbead eye from at least five animals per cohort was used. Retinal and nuclear layer thickness were measured at five different retinal locations per image using the Measurement tool in ImagePro. For RGC counts, the number of CTB-positive and DAPI-positive cells within the ganglion cell layer were counted using the Count tool in Photoshop. At least eight images per animal were quantified to determine an average retinal thickness.

**Table 1:** **Primary antibodies used for brain and whole eye immunohistochemistry**.

**Protein**	**Dilution used**	**Catalog number**	**Vendor**
Alzheimer precursor protein (APP)	1:50	MAB348	1
β-amyloid (Aβ)	1:200	#2454	2
Brain derived neurotrophic factor (BDNF)	1:100	NBP2-42215	3
CD44	1:150	NB600-1317	3
C1q	1:1000	A301	4
Ceruloplasmin (Cp)	1:500	#611488	5
Choline acetyltransferase (ChAT)	1:100	AB144P	1
Glial fibrillary acidic protein (GFAP)	1:500	MAB360	1
Interleukin 1β (IL1β)	1:50	AF-501-NA	6
Interleukin 6 (IL6)	1:400	ab6672	7
IL6Rα membrane bound (IL6Rαm)	1:50	sc-600	8
Ionized Ca^2+^-binding adapter molecule 1 (Iba1)	1:400	ab107159	7
NeuN	1:500	NBP1-92693	3
NF-κB	1:100	sc-372	8
p75 neurotrophin receptor (p75)	1:500	G323A	9
Phosphorylated neurofilament-H (pNF-H)	1:1000	801601	10
Tumor necrosis factor α (TNFα)	1:300	AF-510-NA	6

*1, EMD Millipore, Billerica, MA; 2, Cell Signaling Technology, Danvers, MA; 3, Novus Biologicals, Littleton CO; 4, Quidel Corp., San Diego, CA; 5, BD Transductions Labs, San Jose, CA; 6, R and D Systems, Minneapolis, MN; 7, AbCam, Cambridge, MA; 8, Santa Cruz Biotechnologies, Dallas, TX; 9, Promega Corp., Madison, WI; 10, BioLegend, San Diego, CA*.

### Statistical analysis

All data are expressed as mean ± standard error unless indicated otherwise. The number of samples used in each experiment is provided in the appropriate methods description or figure legend. Statistical comparisons between two independent measurements were made using two-sided *t*-tests, following confirmation of normality for each using the Shapiro-Wilk normality test; samples for which normality failed were compared using the Mann-Whitney Rank Sum Test (SigmaPlot 12.5, Systat Software, Inc., Chicago, IL). Comparisons between multiple groups were made using one way analysis of variance (ANOVA) followed by the Holm-Sidak Pairwise Multiple Comparison test (SigmaPlot 11.1, Systat Software, Inc., Chicago, IL). Comparisons of a sample mean to a hypothesized or predicted value were made using a one-sided *t*-test. Actual *p*-values of significance are indicated where appropriate in the results section or figure legends. All comparisons for which significance is reported achieved or exceeded a *post-hoc* calculation of power of 0.80.

## Results

Two rats, one from each HE3286 group, were euthanized before completion of the study due to complications from oral gavage. Treatment with HE3286 for 28 days had few directly observable adverse effects, as rats were active, responsive and showed no overt signs of distress. HE3286 did result in weight loss in both the 20 and 100 mg/kg groups (Table 2). Weights on day 0 (first day of treatment) ranged from 283 to 430 g, and mean weights for the three treatment groups were not significantly different (*p* = 0.523). Weights on day 28 (last day of treatment) ranged from 290 to 440 g, with no significant difference in mean weight between the three treatment groups (*p* = 0.130). However, rats that received vehicle gained 14.3 g during the experimental period, while rats that received 20 mg/kg HE3286 lost 21.2 g and rats that received 100 mg/kg HE3286 lost 38.0 g. Comparing percent change in weight over the course of treatment for each animal showed that HE3286 decreased weight in both the 20 mg/kg group (*p* < 0.001, compared to vehicle) and 100 mg/kg group (*p* < 0.001, compared to vehicle; *p* = 0.042 compared to 20 mg/kg).

**Table 2:** **Weight loss with HE3286 treatment following microbead injection**.

**Treatment group**	**Weight day 0 (g)**	**Weight day 28 (g)**	**% Change (%)**
Vehicle	357.2 ± 19.7	371.5 ± 21.0	+4.03
20 mg/kg HE3286	347.5 ± 6.5	326.3 ± 9.8	−6.16^*^
100 mg/kg HE3286	371.7 ± 15.0	333.7 ± 14.7	−10.26^*^^†^

* p < 0.001 compared to vehicle;

† *p = 0.042 compared to 20 mg/kg dose (n = 6 rats per group)*.

### Oral delivery of HE3286 has no effect on ocular pressure

**Figure 1:**
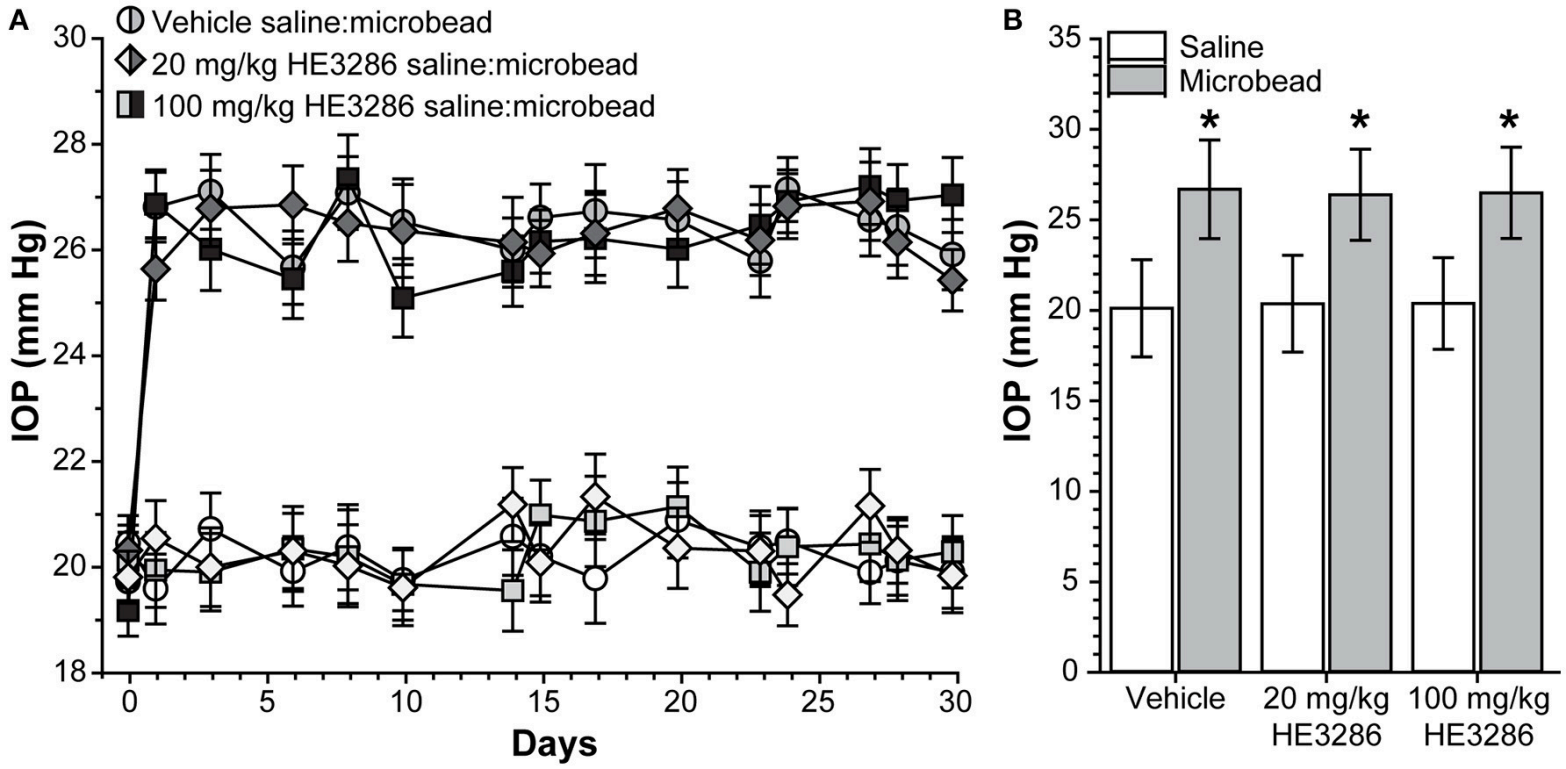
**HE3286 treatment does not lower ocular pressure following microbead occlusion. (A)** Mean intraocular pressure (IOP) in rats before (day 0) and following (days ≥1) a single unilateral injection of polystyrene microbeads (5.0 μl) into the anterior chamber. The fellow eye was injected with an equivalent volume saline. **(B)** Microbead-injected eyes exhibited an increase in IOP following injection (mean ± SEM shown) compared to saline eyes, regardless of treatment. ^*^p ≤ 0.002, *n* = 6 per treatment group.

Baseline IOP measurements ranged from 19.15 ± 0.50 to 20.29 ± 0.55 mm Hg and were similar for all treatment groups (*p* = 0.638). Microbead injection elevated IOP in all eyes for the full 28 day period regardless of treatment (Figure 1A). In vehicle-treated rats, elevation was 32.7% compared to saline-injected eyes: 26.69 ± 2.73 vs. 20.12 ± 2.69 mm Hg; *p* = 0.002 (Figure 1B). Similar increases of 29.6% and 30.0% were observed for the 20 mg/kg HE3286-treated group (26.39 ± 2.51 vs. 20.37 ± 2.68 mm Hg, *p* = 0.002) and for the 100 mg/kg HE3286-treated group (26.49 ± 2.52 vs. 20.39 ± 2.53 mm Hg, *p* = 0.002), respectively. Mean IOP of saline eyes did not significantly differ for any treatment group (*p* = 0.997); the same was true for microbead eyes (*p* = 0.997).

### HE3286 rescues anterograde axonal transport to the superior colliculus

**Figure 2:**
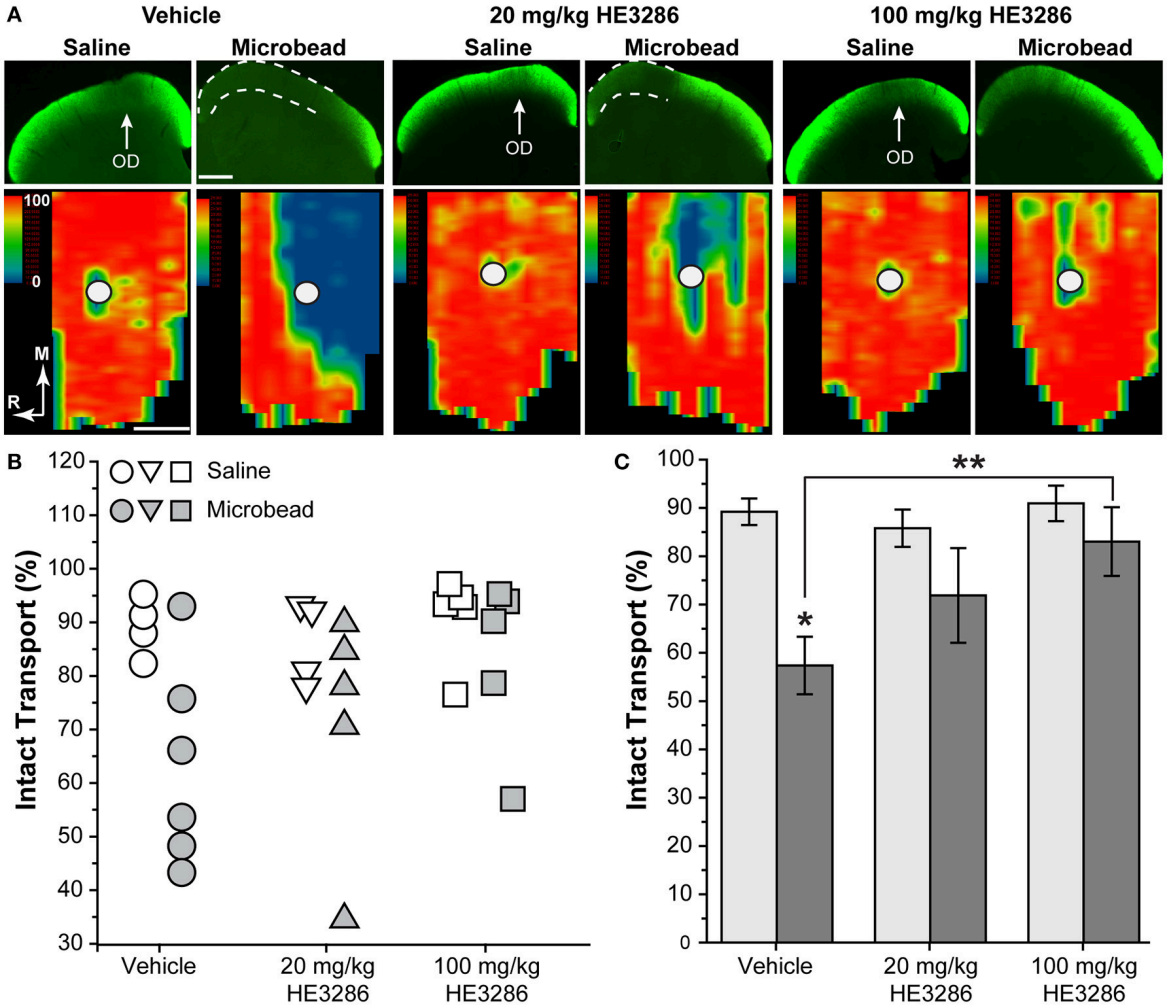
**HE3286 treatment rescues axonal transport following microbead occlusion. (A)** Coronal sections (top row) through the superior colliculus following intravitreal injection of CTB (green) into saline- and microbead-injected eyes of vehicle- and HE3286-treated rats. Deficits in anterograde transport of CTB (dotted lines) due to microbead-induced elevations in IOP are most apparent in vehicle rats. Representation of the optic disc in the retina (OD) lacks transport due to absence of RGCs. Retinotopic maps (bottom row) reconstructed from serial sections of SC with optic disc indicated (circles). Density of the transported CTB signal ranges from 0% (blue) to 50% (green) to 100% (red). Medial (M) and rostral (R) orientations are indicated. Scale: 500 μm. **(B)** Intact transport for individual saline- and microbead-injected eyes per treatment group given as fraction of SC retinotopic map with CTB signal ≥70% of the maximum. Two SCs from saline-injected eyes of vehicle-treated rats and one SC from a saline-injected 20 mg/kg HE3286-treated rat were excluded from analysis due to lack of CTB uptake by RGC. **(C)** Mean level of intact transport in SC was reduced by elevated IOP in vehicle group compared to saline eye (^*^p = 0.003). Higher dose of HE3286 prevented this reduction (^**^p = 0.025). *n* = 4–6 animals per treatment group.

Deficits in anterograde axonal transport from retina to SC are an early sign of pathogenesis in rodent models of glaucoma, including microbead occlusion (Crish et al., 2010; Lambert et al., 2011; Dapper et al., 2013; Ward et al., 2014). Figure 2 compares the level of intact transport of fluorescently labeled CTB to the SC of vehicle-, 20 mg/kg HE3286-, and 100 mg/kg HE3286-treated rats. Deficits in transport are readily apparent in the SC from microbead-injected eyes of vehicle rats (dotted lines, Figure 2A top row). These deficits tend to fill in complete retinotopic sectors when reconstructed from serial sections through the SC (Figure 2A bottom row), consistent with our previous studies (Lambert et al., 2011; Dapper et al., 2013). Variability in transport was low in SC from saline-injected eyes (Figure 2B), which had similarly intact transport for all groups: 89.2 ± 2.7% for vehicle, 85.8 ± 3.9% for 20 mg/kg HE3286, and 90.9 ± 3.7% for 100 mg/kg HE3286 (Figure 2C; *p* = 0.593). Elevated IOP due to microbead injection decreased intact transport to the SC in vehicle-treated rats to 57.4 ± 6.0% intact transport (Figure 2C; *p* = 0.003 compared to saline). Treatment with HE3286 attenuated transport deficits dramatically, as intact transport to the SC from microbead-injected eyes was 71.9 ± 9.8 and 83.0 ± 7.1% for the 20 and 100 mg/kg HE3286 treatment groups, respectively (Figure 2C); neither differed from intact transport from the saline eye (*p* ≥ 0.270). Compared to SC from microbead-injected eyes in the vehicle group, the improvement was significant for the 100 mg/kg HE3286 cohort (*p* = 0.025). No difference in SC volume was detected for any groups or any treatments (*p* = 0.082).

### HE3286 counters the influence of elevated IOP throughout the optic projection

#### Brain-derived neurotrophic factor (BDNF)

**Figure 3:**
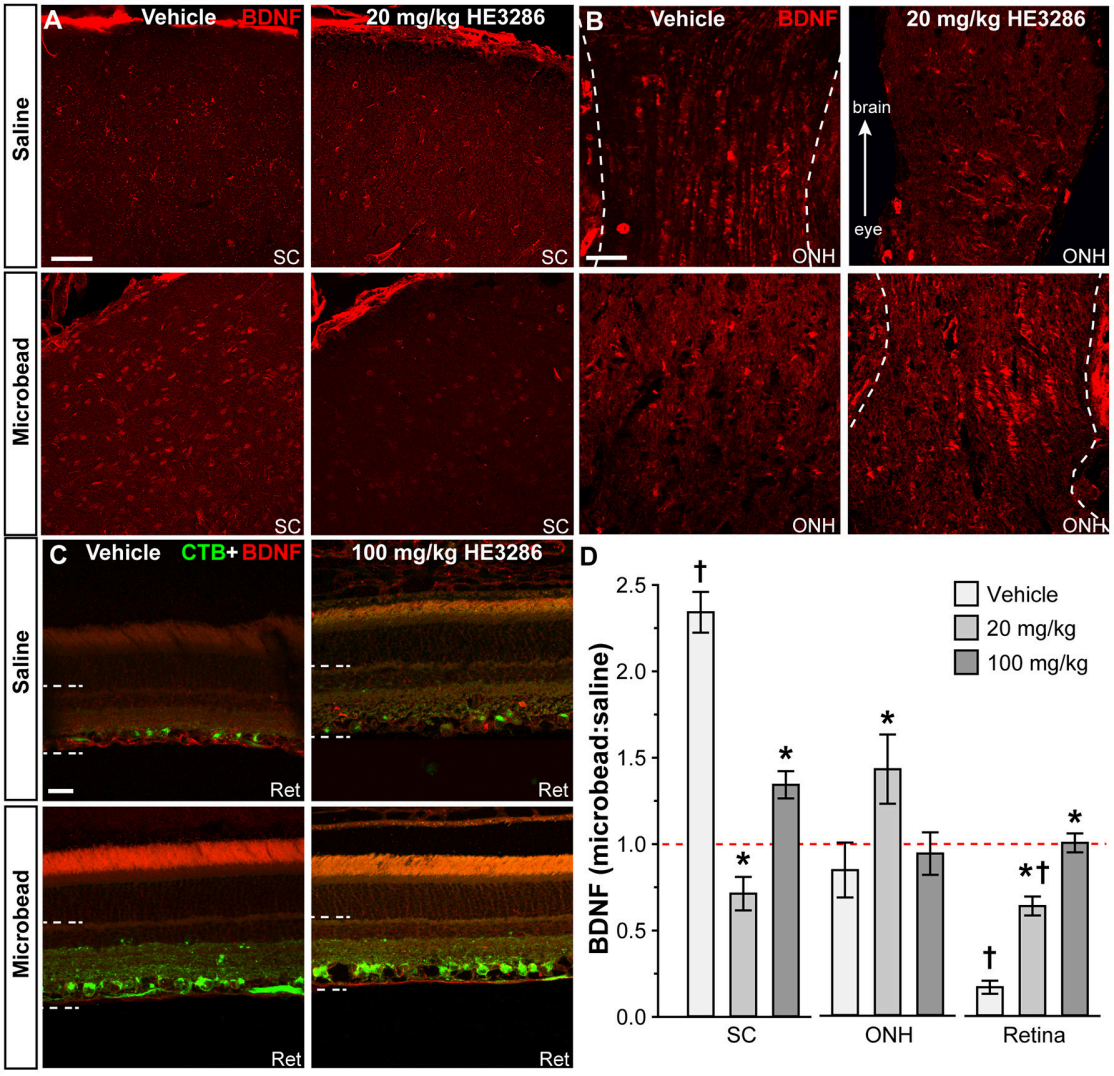
**HE3286 treatment influences BDNF levels in optic projection**. Confocal micrographs of saline- and microbead-eye tissue from vehicle and HE3286 rats show immunolabeling for BDNF in superior colliculus (SC, **A**), optic nerve head (ONH, **B**), and retina (Ret, **C**), which also shows RGCs labeled by CTB uptake. Dotted lines indicate region of retina where immunolabel was quantified. Scale: 50 μm **(A,B)** and 20 μm **(C). (D)** Bar graphs indicate quantification of levels of BDNF in superior colliculus (SC), optic nerve head (ONH), and retina expressed as the microbead:saline ratio for vehicle and HE3286 treatment groups. ^†^Indicates significant departure from expected ratio of unity (*p* ≤ 0.02), which is indicated (dotted line). ^*^indicates *p* ≤ 0.042 compared to ratio for vehicle group. *n* = 6 animals per vehicle treatment group, 5 animals per 20 mg/kg and 100 mg/kg HE3286 groups for SC; *n* = 3 animals per treatment group for ONH and retina analysis.

Deficits in anterograde transport to the SC of glaucomatous mice is accompanied by significant changes in local neurochemistry, including increased localization of BDNF (Crish et al., 2013). Within the SC of vehicle rats, BDNF also appeared to increase with microbead-induced elevated IOP (Figure 3A, left); treatment with HE3286 (20 mg/kg) appeared to blunt this increase (Figure 3A, right). In the optic nerve head (ONH; Figure 3B), which is a major site of stress in glaucoma (Hernandez, 2000), levels of BDNF appeared similar in saline- and microbead-injected eyes from vehicle treated rats. Treatment with HE3286 (20 mg/kg) appeared to increase BDNF in the ONH of microbead-injected eyes compared to saline. Finally, levels of BDNF in the retina decreased following microbead injection in vehicle-treated rats, with HE3286 again blunting this effect (Figure 3C).

When quantified (Figure 3D), the ratio of BDNF in microbead to saline SC for vehicle rats was significantly greater than an expected ratio of unity: 2.34 ± 0.12 (*p* < 0.001). This ratio was diminished by 70% compared to vehicle in the 20 mg/kg HE3286 group (0.71 ± 0.09; *p* < 0.001) and by 43% in the 100 mg/kg group (1.34 ± 0.08; *p* < 0.001). In the ONH (Figure 3D, middle), the ratio of microbead to saline BDNF increased with 20 mg/kg HE3286 by 69%: 1.43 ± 0.20 vs. 0.84 ± 0.16 for vehicle (*p* = 0.042). The 34% increase in this ratio for the 100 mg/kg dose was not significant compared to vehicle (0.93 ± 0.12; *p* = 0.66). Finally, in the retina, treatment with HE3286 increased the microbead:saline ratio of BDNF by 282% in the 20 mg/kg group (0.64 ± 0.05) and by 501% in the 100 mg/kg group (1.00 ± 0.05) when compared to vehicle (0.17 ± 0.04; *p* < 0.002); the 100 mg/kg HE3286 ratio was significantly below an expected ratio of unity (*p* = 0.02). Thus, as microbead-induced elevations in IOP degrade anterograde transport in the optic projection, HE3286 treatment generally has the effect of opposing the change in BDNF levels induced by elevated IOP.

Note that for BDNF and all other immunolabeled proteins we quantified, the total SC area for saline (7.013 ± 0.006 mm^2^) and microbead (7.017 ± 0.003 mm^2^) eyes or vehicle (7.019 ± 0.002 mm^2^) and HE3286 groups (7.012 ± 0.005 mm^2^) did not vary (*p* ≥ 0.088). Similarly, total area quantified in the nerve head did not vary between saline (7.019 ± 0.002 mm^2^) and microbead (7.022 ± 0.002 mm^2^) eyes or between vehicle (7.018 ± 0.003 mm^2^) and HE3286 groups (7.022 ± 0.002 mm^2^; *p* > 0.279). Finally, total area quantified in the retina was similar between saline (2.756 ± 0.203 mm^2^) and microbead (2.712 ± 0.198 mm^2^) eyes or between vehicle (2.996 ± 0.204 mm^2^) and HE3286 (2.603 ± 0.199 mm^2^; *p* > 0.076) groups.

#### Microglia activation

**Figure 4:**
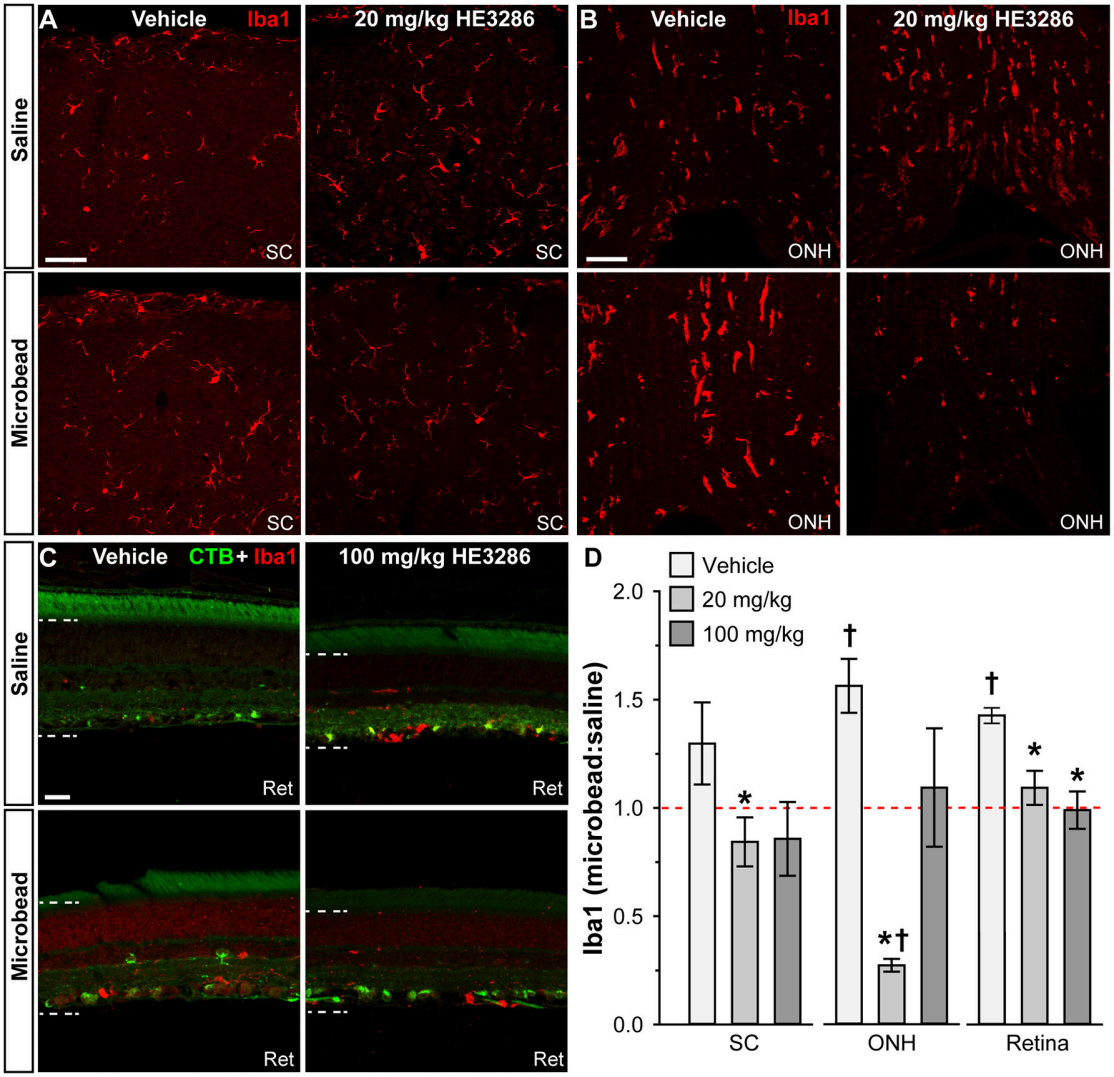
**HE3286 treatment influences Iba1-labeled microglia in optic projection**. Confocal micrographs of saline- and microbead-injected tissue from vehicle and HE3286 rats show immunolabeling for the microglia marker Iba1 in superior colliculus (SC, **A**), optic nerve head (ONH, **B**), and retina (Ret, **C**), which also shows RGCs labeled by CTB uptake. Dotted lines indicate region of retina where immunolabel was quantified. Scale: 50 μm **(A,B)** and 20 μm **(C)**. **(D)** Bar graphs indicate quantification of Iba1 in superior colliculus (SC), optic nerve head (ONH), and retina expressed as the microbead:saline ratio for vehicle and HE3286 treatment groups. ^†^ indicates significant departure from expected ratio of unity (*p* ≤ 0.045), which is indicated (dotted line). ^*^Indicates *p* ≤ 0.037 compared to ratio for vehicle group. *n* = 6 animals per vehicle treatment group, 5 animals per 20 mg/kg and 100 mg/kg HE3286 groups for SC; *n* = 3 animals per treatment group for ONH and retina analysis.

Given HE3286's proposed anti-inflammatory action and the contribution of microglia as neuroinflammatory mediators in glaucoma, we examined how treatment influences levels of ionized calcium-binding adapter molecule 1 (Iba1), a microglia-specific marker (Ito et al., 1998; Soto and Howell, 2014; Mac Nair and Nickells, 2015). In the SC, microbead-induced elevated IOP elicited a modest increase in Iba1 levels in vehicle animals, which was mitigated by HE3286 (Figure 4A). In the ONH, elevated IOP sharply increased Iba1, which again was prevented in the HE3286 group (Figure 4B). Iba1 appeared elevated in microbead retina from vehicle-treated rats, while treatment with HE3286 had the opposite effect (Figure 4C). The influence of HE3286 was significant in all three tissues (Figure 4D). Treatment with the 20 mg/kg dose of HE3286 reduced the ratio of Iba1 in microbead to saline SC by 63%: 0.84 ± 0.11 compared to 1.29 ± 0.19 for vehicle (*p* = 0.037). The 100 mg/kg dose had a similar influence (0.86 ± 0.17, *p* = 0.061). The microbead:saline ratio of Iba1 in ONH for vehicle rats was significantly greater than an expected ratio of unity: 1.56 ± 0.12 (*p* = 0.045). This ratio was diminished by 83% in the 20 mg/kg HE3286 group (0.27 ± 0.03; *p* = 0.005); 100 mg/kg HE3286 had far less effect (1.09 ± 0.27; *p* = 0.193). For vehicle retina, the Iba1 microbead to saline ratio was significantly more than 1 (1.42 ± 0.04; *p* = 0.007). Treatment with both 20 mg/kg and 100 mg/kg HE3286 decreased this ratio compared to vehicle: 1.09 ± 0.08 for the 20 mg/kg group (*p* = 0.018) and 0.99 ± 0.07 (*p* = 0.009) for the 100 mg/kg group.

#### Markers of neuroinflammation or neurodegeneration

**Figure 5:**
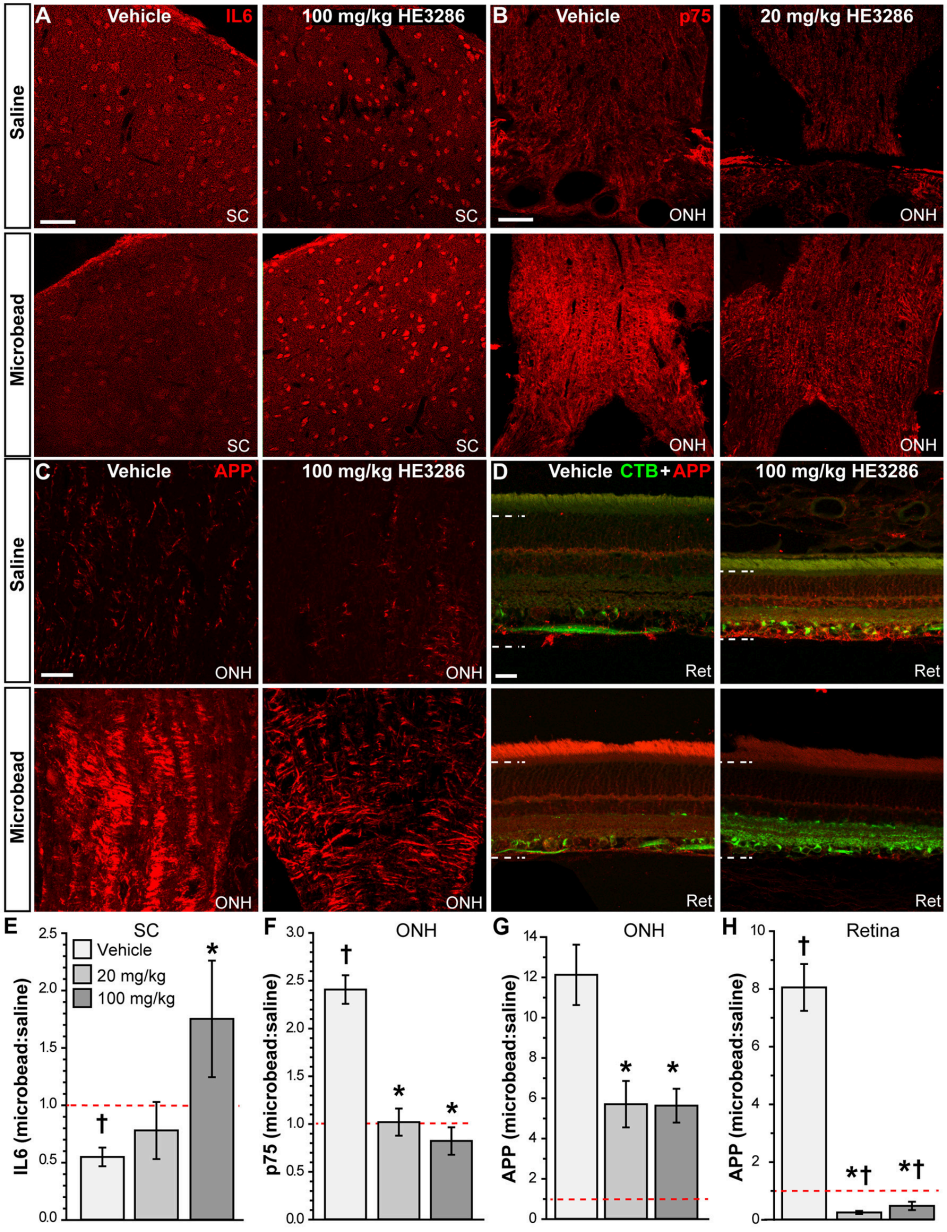
**HE3286 treatment influences other degenerative markers in optic projection**. Confocal micrographs of saline- and microbead-injected tissue from vehicle and HE3286 rats show immunolabeling for IL6 in superior colliculus (SC, **A**), p75 in optic nerve head (ONH, **B**), and APP in optic nerve head (ONH, **C**) and retina (Ret, **D**), which also shows RGCs labeled by CTB uptake. Dotted lines indicate region of retina where immunolabel was quantified. Scale: 50 μm **(A–C)** and 20 μm **(D)**. Bar graphs **(E–H)** indicate quantification of each of these markers in the specified tissue expressed as the microbead:saline ratio for vehicle and HE3286 treatment groups. SC: superior colliculus; ONH: optic nerve head. ^†^Indicates significant departure from expected ratio of unity (*p* ≤ 0.047), which is indicated (dotted line). ^*^indicates *p* ≤ 0.027 compared to ratio for vehicle group. *n* = 6 animals per vehicle treatment group, 5 animals per 20 mg/kg and 100 mg/kg HE3286 groups for SC; *n* = 3 animals per treatment group for ONH and retina analysis.

Other significant markers implicated in neuroinflammation or neurodegeneration were also modulated in the optic projection by HE3286. Interleukin 6 (IL6) is a pro-inflammatory cytokine that can promote RGC survival (Matousek et al., 2012; Song et al., 2013). In the SC of vehicle rats, IL6 localization appeared to diminish with elevated IOP; this was reversed with HE3286 (Figure 5A). The p75 neurotrophin receptor is a member of the tumor necrosis factor receptor family and has diverse roles in neuronal activity, plasticity, and injury response (Meeker and Williams, 2015). We found that p75 levels increased within the ONH of microbead eyes from vehicle rats; treatment with 20 mg/kg HE3286 prevented this increase (Figure 5B). Finally, amyloid precursor protein (APP) is cleaved to form β-amyloid, a major component of amyloid plaques. Recent evidence suggests this protein and its cleaved products may be involved in the pathogenesis of glaucoma (Jain and Aref, 2015). HE3286 appeared to increase APP levels in saline-injected eyes (Figure 5D). We found that microbead-induced elevations in IOP increased APP in the optic nerve head (Figure 5C) and retina (Figure 5D) of vehicle rats. In both tissues, HE3286 prevented this increase.

When quantified (Figure 5E), the ratio of IL6 in microbead to saline SC was significantly less than an expected value of unity: 0.55 ± 0.08 (*p* = 0.005). This trend was reversed by 100 mg/kg HE3286, which increased the IL6 ratio by 219% compared to vehicle: 1.75 ± 0.51 (*p* = 0.047). In the ONH (Figure 5F), the microbead to saline ratio for p75 was also significantly higher than unity: 2.41 ± 0.15 (*p* = 0.011). Treatment with both 20 mg/kg and 100 mg/kg HE3286 prevented the increase with elevated IOP, reducing the ratio by 58% (1.02 ± 0.14; *p* = 0.002) and 66% (0.82 ± 0.14; *p* = 0.001), respectively, compared to vehicle. For APP in ONH (Figure 5G), treatment with 20 mg/kg HE3286 decreased the microbead to saline ratio by 53% compared to vehicle: 5.71 ± 1.15 vs. 12.13 ± 1.49 (*p* = 0.027). Similarly, 100 mg/kg HE3286 elicited a 54% decrease to 5.63 ± 0.84 (*p* = 0.019). In the retina (Figure 5H), elevated IOP significantly increased the microbead to saline ratio for APP well above an expected ratio of unity: 8.05 ± 0.81 (*p* = 0.013). Treatment with HE3286 significantly decreased the ratio by 97 and 94% for the 20 mg/kg and 100 mg/kg doses, respectively, compared to vehicle (*p* < 0.001). Both the ratio for 20 mg/kg HE3286 (0.25 ± 0.05) and 100 mg/kg HE3286 (0.48 ± 0.14) were significantly lower than unity (*p* ≤ 0.035).

As with BDNF (Figure 3) and Iba1 (Figure 4), in each of the cases illustrated in Figure 5, treatment with one or both doses of HE3286 tended to oppose the action of elevated IOP in the vehicle cohort, as indicated by the directionality of the change in the microbead:saline ratio for each. If the ratio >1 in vehicle tissue, HE3286 treatment pushed the ratio below unity; if the vehicle ratio <1, HE3286 increased the ratio above unity. This tendency held for other proteins we tested in SC, ONH, and retina and for which HE3286 elicited a significant change in expression level. These results are summarized in Table 3.

**Table 3:** **Significant HE3286-induced changes in expression compared to vehicle**.

**Target**	**Tissue**	**Vehicle (*p*-value)**	**20 mg/kg**	**100 mg/kg**	***p*-values**
TNFα	ONH	1.68 ± 0.20 (0.038)	0.28 ± 0.10: 83%↓	No change	0.003
IL6Rαm	ONH	1.93 ± 0.09 (<0.001)	0.72 ± 0.03: 62%↓	0.74 ± 0.11: 63%↓	<0.001
IL6	Retina	5.43 ± 0.84 (0.006)	2.10 ± 0.37: 61%↓	2.55 ± 0.65: 53%↓	≤0.045
p75	Retina	2.41 ± 0.22 (0.022)	1.53 ± 0.20: 37%↓	1.26 ± 0.21: 48%↓	≤0.041
CD44	Retina	1.32 ± 0.10	No change	0.48 ± 0.11: 63%↓	0.005
Aβ	Retina	8.89 ± 0.58 (<0.001)	13.19 ± 1.33: 48%↑	5.02 ± 0.88: 44%↓	≤0.042

*Tissue expression given as microbead:saline, mean ± SEM (n = 3 animals per groups). Vehicle p-value indicates significance for ratio compared expected value of unity. ONH, optic nerve head; IL6, interleukin-6; p75, low-affinity neurotrophin receptor; CD44, homing cell adhesion glycoprotein; TNFα, tumor necrosis factor α; IL6Rαm, membrane bound IL6 receptor α; Aβ, beta-amyloid. ↑, increase compared to vehicle; ↓, decrease compared to vehicle*.

### HE3286 modulates common pathogenic markers for glaucoma in the retina

**Figure 6:**
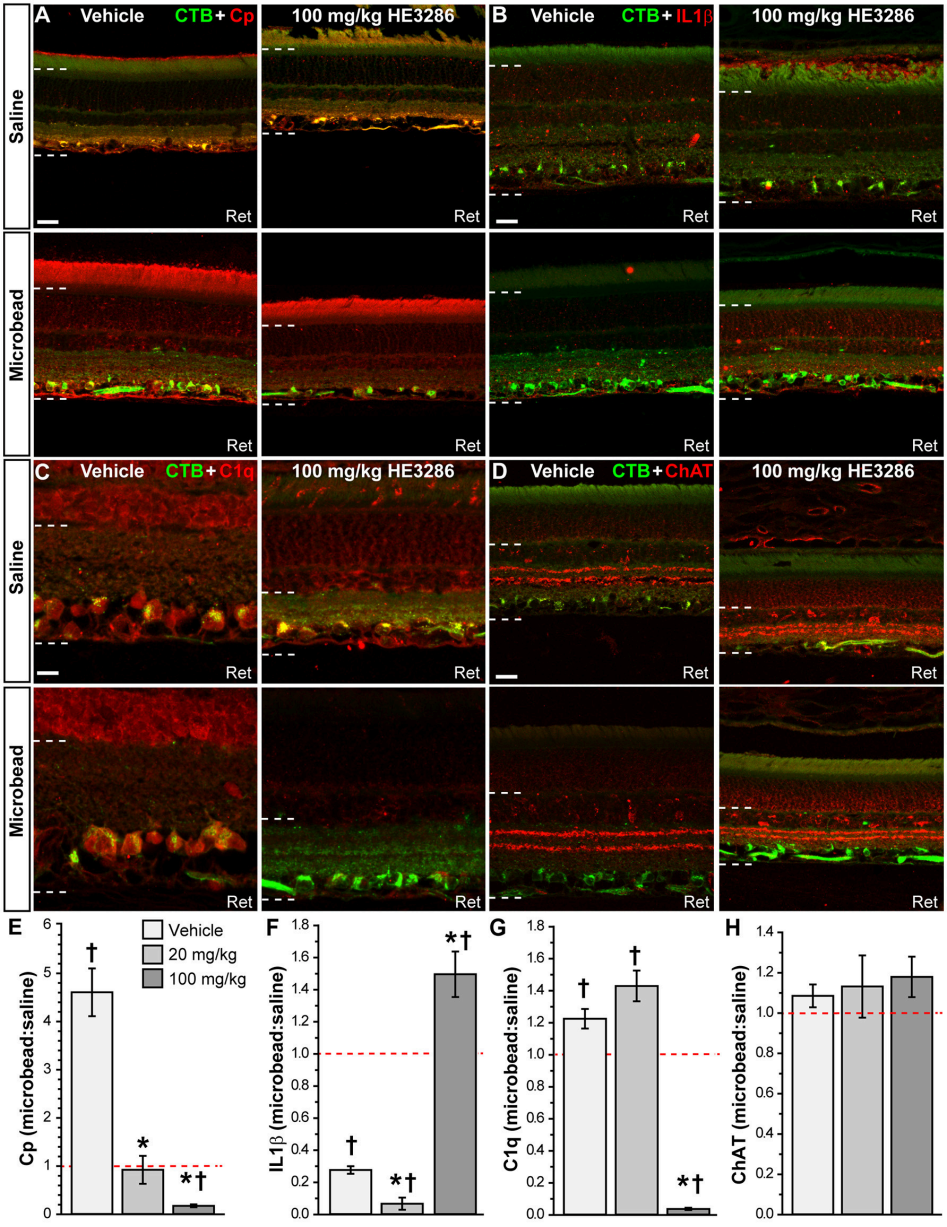
**HE3286 treatment influences retinal expression of common pathogenic markers**. Confocal micrographs of saline- and microbead-injected from vehicle and HE3286 rats show immunolabeling in retina (Ret) for ceruloplasmin (Cp, **A**), interleukin 1β (IL1β, **B**), and C1q, the first component of the classical complement pathway **(C)**. Localization of amacrine cell neurons specific for ChAT (choline acetyltransferase) is shown for comparison **(D)**. CTB-labeled RGCs are shown in each panel. Dotted lines indicate region of retina where immunolabel was quantified. Scale: 20 μm **(A,B,D)** or 10 μm **(C)**. Bar graphs **(E–H)** indicate quantification of each of these markers expressed as the microbead:saline ratio for vehicle and HE3286 treatment groups. ^†^Indicates significant departure from expected ratio of unity (*p* ≤ 0.037), which is indicated (dotted line). ^*^indicates *p* ≤ 0.033 compared to ratio for vehicle group. *n* = 3 animals per treatment group.

Next we examined how HE3286 treatment influences localization of common markers for neurodegeneration in glaucomatous retina. Ceruloplasmin (Cp) is a positive acute phase protein upregulated during inflammation (Denko, 1979; Goldstein et al., 1979). In vehicle retina, Cp increased dramatically with microbead-induced elevated IOP; HE3286 prevented this increase (Figure 6A). Interleukin 1β (IL1β) is a pro-inflammatory cytokine that can promote neurodegeneration or protection depending on whether the injury is acute or chronic (Matousek et al., 2012; Song et al., 2013). In vehicle retina, IL1β decreased with elevated IOP (Figure 6B, left). While treatment with 20 mg/kg HE3286 further reduced expression (images not shown), 100 mg/kg HE3286 reversed this trend (Figure 6B, right). In vehicle retina, elevated IOP appeared to increase expression of C1q, an early component of the classical complement pathway, especially in CTB-labeled RGCs (Figure 6C, left). This too was prevented by 100 mg/kg HE3286 (Figure 6C, right). Finally, we also tested localization of choline acetyltransferase (ChAT), a marker for cholinergic amacrine cell neurons, to determine if HE3286 affects levels of common proteins not typically associated with glaucoma progression. Neither elevated IOP nor treatment appeared to influence localization levels (Figure 6D), which were consistent with other studies (Kang et al., 2004; Feng et al., 2006; Samuel et al., 2011).

When quantified, the ratio of Cp in microbead to saline retina in the vehicle group was well above an expected value of unity: 4.61 ± 0.49 (*p* = 0.018; Figure 6E). Treatment with HE3286 abated this increase by 80% compared to vehicle in the 20 mg/kg group (0.92 ± 0.29; *p* = 0.003) and by 96% in the 100 mg/kg group (0.18 ± 0.03; *p* < 0.001). For IL1β (Figure 6F), elevated IOP significantly reduced levels compared to saline retina in the vehicle group, yielding a ratio of 0.28 ± 0.02 (*p* = 0.001). Treatment with 20 mg/kg HE3286 further reduced IL1β by 76% (0.07 ± 0.04; *p* = 0.009). However, 100 mg/kg HE3286 had the opposite effect, increasing levels by 440% compared to vehicle (1.49 ± 0.14; *p* = 0.001). Elevated IOP in vehicle rats significantly increased levels of C1q compared to saline retina: 1.225 ± 0.061 (*p* = 0.033, Figure 6G). While 20 mg/kg HE3286 had little effect (1.430 ± 0.096), 100 mg/kg HE3286 significantly reduced the C1q microbead to saline ratio compared to vehicle: 0.037 ± 0.007 (*p* < 0.001). As expected, levels of ChAT were the same in saline and microbead retinas in all treatment groups (Figure 6H, *p* ≥ 0.461).

### HE3286 changes NFκB localization in the optic projection

**Figure 7:**
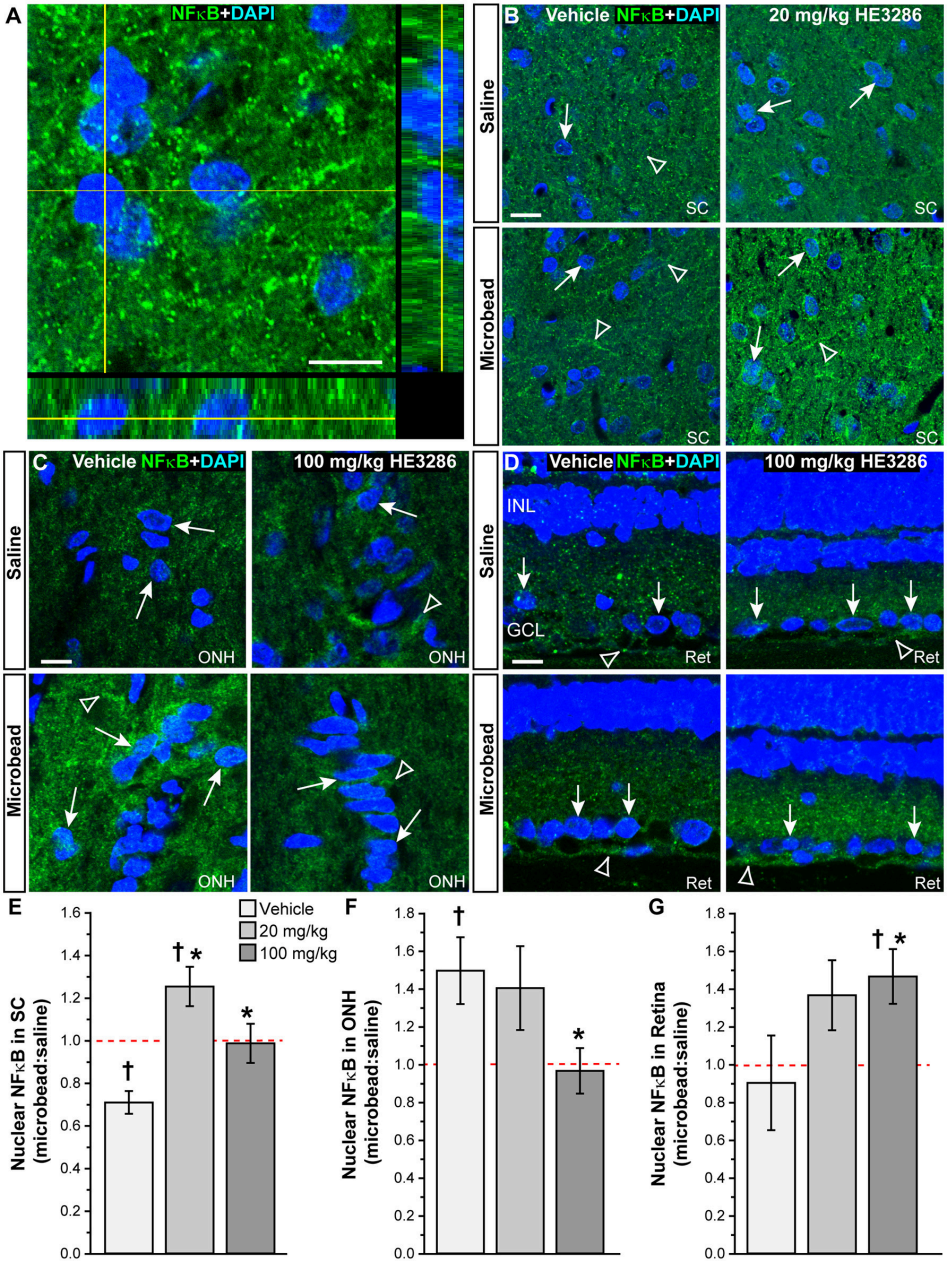
**HE3286 modulates NFκB nuclear localization in the optic projection. (A)** Confocal micrograph of a coronal section through naïve rat SC shows localization of NFκB (green) both outside and within DAPI-labeled nuclei (blue). Lines indicate location of orthogonal rotations through nuclei. Scale: 10 μm. Representative images (single optical plane) of superior colliculus (SC, **B**), optic nerve head (ONH, **C**) and retina (Ret, **D**) from vehicle and HE3286 rats shows localization of NFκB (green) both within (arrows) and external to (arrowhead) DAPI-stained nuclei. INL: inner nuclear layer of retina; GCL: ganglion cell layer. Scale: 10 μm. Bar graphs show nuclear localization of NFκB in SC **(E)**, optic nerve head (ONH, **F**), and retina **(G)** expressed as the ratio of microbead:saline in vehicle and HE3286 treated tissue; ratio of 1, or unity, indicated by dotted line. ^†^Indicates significant departure from expected ratio of unity (*p* ≤ 0.026). ^*^Indicates *p* ≤ 0.044 compared to ratio for vehicle group. *n* = 5 animals per treatment group.

One proposed mechanism of action for HE3286 is to modulate the transcription factor NFκB, which translocates to the nucleus when activated (Offner et al., 2009). Using an antibody with proven efficacy in brain (Herkenham et al., 2011; Figure 7A), we examined and quantified the degree of nuclear localization of NFκB in SC, optic nerve head, and retina. In vehicle-treated rats, NFκB localized both within and external to nuclei in SC; microbead SC appeared to have less nuclear localization (Figure 7B). Treatment with HE3286 increased NFκB particularly in microbead SC. Within the ONH (Figure 7C), microbead-induced elevations in IOP increased NFκB in nuclei for the vehicle group; this was prevented with HE3286 treatment. In retinal sections (Figure 7D), elevated IOP had little effect on NFκB localization. However, HE3286 appeared to increase NFκB translocation, particularly in RGCs of microbead retina.

Using an established algorithm (Carmona et al., 2007; Schneider et al., 2012), we quantified the degree of nuclear localization of NFκB as the ratio of levels in tissue from microbead to saline eyes (Figures 7E–G). For SC (Figure 7E), this ratio was significantly lower than an expected value of 1 for vehicle rats (*p* = 0.006), consistent with our qualitative observation (Figure 7B). Treatment with HE3286 reversed this trend as we found that the ratio of microbead to saline nuclear NFκB increased 77% (1.25 ± 0.09) in rats treated with 20 mg/kg HE3286 and by 39% (0.99 ± 0.09) in rats treated with 100 mg/kg HE3286; both changes were significant compared to vehicle (0.71 ± 0.5; *p* ≤ 0.031). In the ONH (Figure 7F), NFκB in nuclei increased significantly with microbead elevations in IOP for the vehicle group (1.50 ± 0.18; *p* = 0.047). Treatment with 100 mg/kg HE3286 decreased the ratio of microbead to saline levels by 35% (0.97 ± 0.12), which was significant compared to vehicle (*p* = 0.038). Treatment with 20 mg/kg HE3286 had little effect compared to vehicle (1.41 ± 0.22; *p* = 0.753). Similar to the SC, nuclear NFκB in the retina increased with IOP elevation for the HE3286 treatment groups (Figure 7G). Treatment with 100 mg/kg HE3286 caused a 62% increase (1.47 ± 0.14) compared to the microbead:saline ratio in vehicle (0.90 ± 0.25; *p* = 0.044); this was also significantly different from an expected ratio of 1 (*p* = 0.031). The 20 mg/kg HE3286 dose also increased NFκB nuclear labeling in microbead retina (1.368 ± 0.185), but the 51% increase was not significant both compared to vehicle (*p* = 0.087) and to an expected ratio of unity (*p* = 0.117).

**Figure 8:**
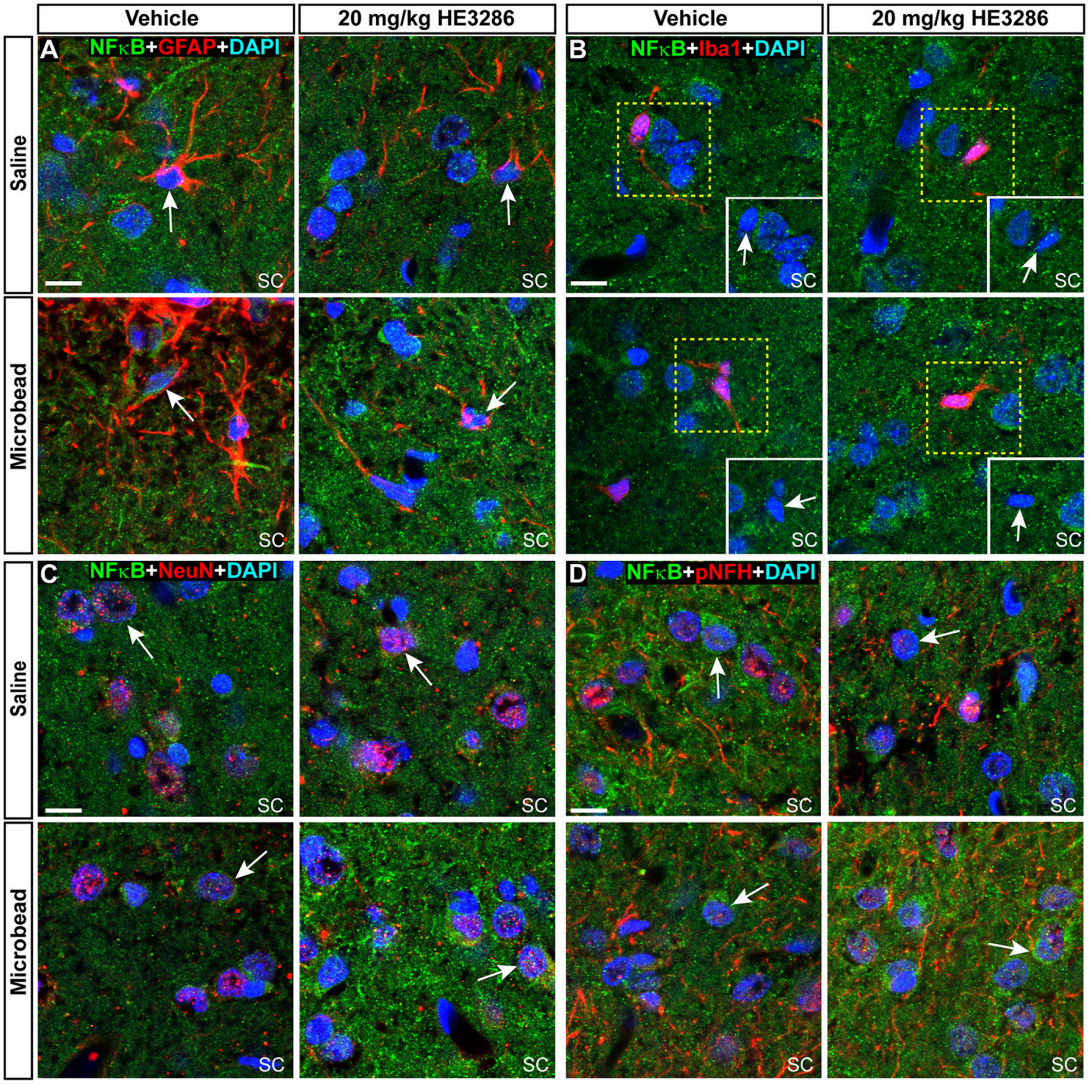
**NFκB localizes to both neuronal and glial nuclei in superior colliculus**. Representative confocal images of NFκB in the superior colliculus (SC) of vehicle- and HE3286-treated rats with all nuclei indicated (DAPI). Sections were also labeled for astrocytes (**A**, GFAP), microglia (**B**, Iba1), or neurons using antibodies against either NeuN **(C)** or phosphorylated neurofilament heavy (pNFH; **D**). Representative nuclei from each cell class demonstrated NFκB localization (arrows). Insets (solid white, **B**) show region contained within dashed box with red channel removed to better visualize nuclear localization of NFκB. Scale: 10 μm.

NFκB activation can promote degeneration or survival depending on the cell type (e.g., glia vs. neurons; Mattson and Camandola, 2001). In SC from each treatment group, NFκB localized to nuclei of GFAP-labeled astrocytes (Figure 8A), Iba1-labeled microglia (Figure 4B), and neurons labeled by NeuN or pNFH (Figures 8C,D). Whether nuclear localization was glial or neuronal did not appear to be influenced by elevated IOP in the vehicle cohort, since SC from saline and microbead eyes showed both. NFκB localization in neuronal nuclei did appear to increase with elevated IOP in HE3286-treated rats, consistent with the overall increase in NFκB we observed earlier (Figures 7B,E).

**Figure 9:**
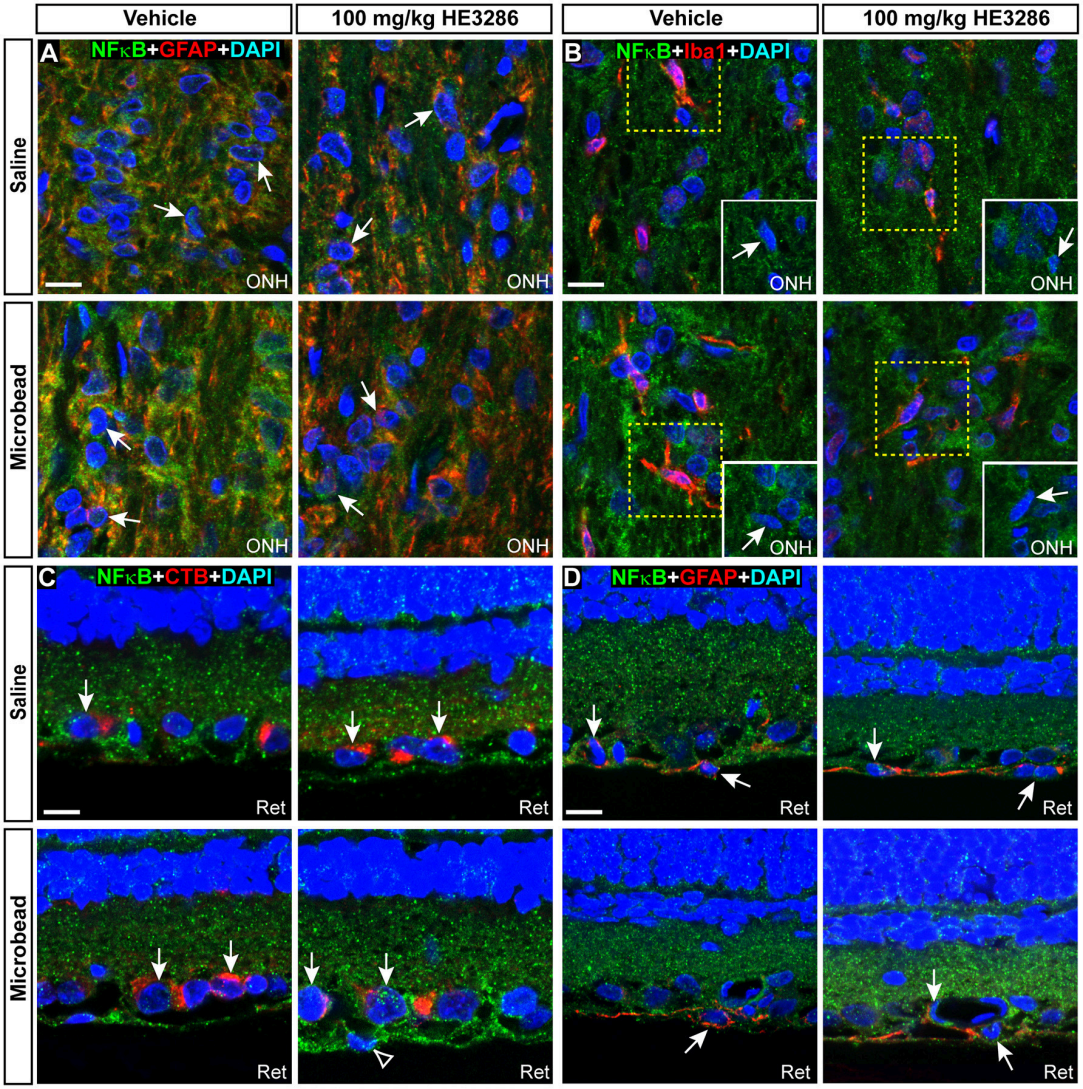
**NFκB localizes to glial nuclei in optic nerve head and to glial and neuronal nuclei in retina**. Representative confocal images of NFκB (green) localization in DAPI-stained nuclei of astrocytes (**A**, GFAP) and microglia (**B**, Iba1) in optic nerve head (ONH) of vehicle- and HE3286-treated rats. Inset in **(B)** is region contained within dashed box with red channel removed to better visualize nuclear localization of NFκB. In retina **(C,D)**, NFκB localizes to nuclei of RGCs with CTB uptake and GFAP-labeled astrocytes within the retina (Ret). Arrows indicate cells positively-identified by specific label. Scale: 10 μm.

Within the ONH, the majority of NFκB appeared to localize in the cytoplasm surrounding DAPI-labeled nuclei, though nuclei of some astrocytes (Figure 9A) and microglia (Figure 9B) contained NFκB. Consistent with the overall increase within the ONH (Figures 7C,F), microbead-induced elevated IOP appeared to increase glial NFκB nuclear localization in vehicle rats, which was attenuated by HE3286. In retina, nuclei of RGCs identified by CTB uptake and GFAP-labeled astrocytes also demonstrated NFκB localization (Figures 9C,D). Elevated IOP appeared to decrease nuclear NFκB in vehicle rats, with HE3286 treatment reversing this trend. Again, this finding is consistent with the overall trend of NFκB localization we quantified in retina (Figures 7D,G).

### HE3286 decreased retinal thickness in rats with induced ocular hypertension

**Figure 10:**
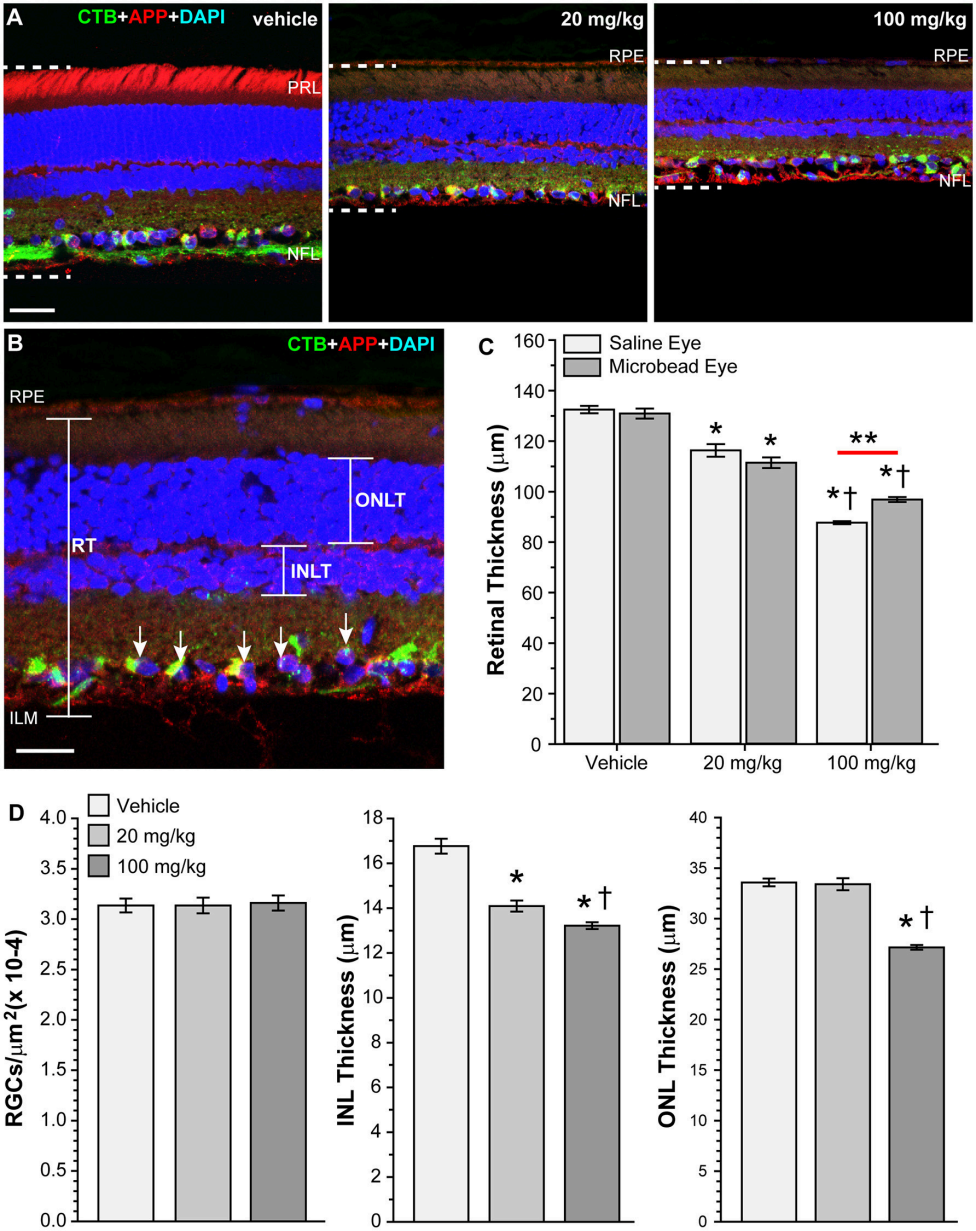
**HE3286 treatment decreases retinal thickness. (A)** Confocal images of vertical retinal sections from vehicle- and HE3286-treated rats showing CTB uptake (green), immunolabeling for APP (red), and DAPI-labeled nuclei (blue). All images were captured in the mid-peripheral region of the retina. Dotted lines show retinal thickness from nerve fiber layer (NFL) to the retinal pigmented epithelial layer (RPE). Scale bar: 30 μm. PRL: photoreceptor layer. **(B)** Higher magnification of retinal section with same markers demonstrating how total retinal thickness (RT), inner nuclear layer thickness (INLT) and outer nuclear layer thickness (ONLT) were measured. RGCs positive for CTB and DAPI (arrows) were counted. Scale bar: 20 μm. ILM: inner limiting membrane. **(C)** Bar graph showing HE3286 treatment decreased retinal thickness. ^*^p < 0.005 compared to vehicle; ^†^*p* < 0.003 compared to 20 mg/kg HE3286; ^**^p = 0.0013; *n* = 8 images per animal, three animals per treatment group. **(D)** Bar graphs showing HE3286 did not reduce RGC number, but did result in thinning of INL and ONL. ^*^p < 0.001 compared to vehicle; ^†^*p* < 0.029 compared to 20 mg/kg HE3286 for INL thickness. ^*^p < 0.001 compared to vehicle; ^†^*p* < 0.001 compared to 20 mg/kg HE3286 for ONL thickness. *n* = 8 images per animal, three animals per treatment group.

While examining vertical retinal sections following immunohistochemistry, we noted what appeared to be a decrease in retinal thickness within the mid-peripheral region of the retina in rats treated with HE3286 compared to vehicle (Figure 10A). To examine this further, we measured retinal thickness across groups and retinal locations. Comparing mean retinal thickness (Figure 10C) showed that for vehicle-treated and 20 mg/kg HE3286-treated rats, retinal thickness was decreased in microbead-injected eyes compared to saline-injected eyes, but this difference was not significant (vehicle: 132.51 ± 1.47 vs. 130.93 ± 1.99 μm, *p* = 0.557; 20 mg/kg dose: 116.35 ± 2.50 vs. 111.47 ± 2.11 μm, *p* = 0.21). In contrast, rats treated with 100 mg/kg HE3286 had significantly thinner retinas in saline-injected eyes (87.70 ± 0.59 μm) compared to microbead-injected eyes (96.91 ± 0.98 μm; *p* = 0.001). Comparing saline-injected eyes across the groups showed that treatment with HE3286 decreased retinal thickness 12–34% compared to treatment with vehicle (*p* < 0.005). Similarly, retinal thickness decreased 15–26% in microbead eyes treated with HE3286 compared to vehicle (*p* < 0.002). Retinal thinning was dependent on dosage, as a 13–25% decrease was observed in eyes that received 100 mg/kg HE3286 compared to 20 mg/kg (*p* < 0.003). To determine if retinal thinning involved neuronal loss, we counted RGCs that were positive for CTB uptake and DAPI nuclear labeling and measured the thickness of the inner nuclear and outer nuclear layers of the retinas (Figure 10D). Treatment with HE3286 did not reduce the number of RGCs in 20 mg/kg (3.14 × 10^−4^ ± 7.77 × 10^−6^ RGCs/μm^2^) or 100 mg/kg (3.16 × 10^−4^ ± 7.48 × 10^−6^ RGCs/μm^2^) HE3296-treated retinas compared to vehicle-treated retinas (3.14 × 10^−4^ ± 6.91 × 10^−6^ RGCs/μm^2^; *p* > 0.963). In contrast, HE3286 treatment reduced INL thickness 16% for the 20 mg/kg dose (14.1 ± 0.2 μm) and 21% for the 100 mg/kg dose (13.2 ± 0.1 μm) compared to vehicle (16.8 ± 0.3 mm; *p* < 0.001). This response was dose-dependent, as 100 mg/kg HE3286 reduced INL thickness a further 6% compared to 20 mg/kg (*p* = 0.029). A similar reduction in ONL thickness was observed at the higher dose of HE3286 (27.1 ± 0.2 μm) compared to vehicle (33.6 ± 0.4 μm) or the 20 mg/kg dose (33.4 ± 0.6 μm; *p* > 0.001)

## Discussion

In this study, we examined the therapeutic potential of a sterol derivative, HE3286, in an inducible model of glaucoma. Daily oral gavage with either 20 mg/kg or 100 mg/kg HE3286 did not affect IOP following microbead or saline injection. Even so, treatment with HE3286 did have tremendous influence on outcomes typically associated with IOP-related axonopathy. We found that HE3286 (1) preserved anterograde axonal transport of CTB from the retina to the superior colliculus in microbead-injected eyes, (2) countered the influence of elevated IOP throughout the optic projection with respect to levels of BDNF, Iba1 and other proteins associated with neuroinflammation and neurodegeneration, (3) modulated pathogenic markers of glaucoma within the retina, (4) increased NFκB localization to neuronal nuclei in the superior colliculus and retina, and (5) decreased NFκB localization to glial nuclei in the optic nerve head.

HE3286 treatment also resulted in weight loss and retinal thinning. In a safety and pharmacokinetic study, Ahlem et al. found that HE3286 had no adverse effects in chronic toxicity studies and was not immunosuppressive (Ahlem et al., 2011). Male rats that received daily oral gavage of 100 to 400 mg/kg HE3268 consumed less food and gained less weight than rats treated with vehicle, but showed no corresponding pathology or other effects on general health (Ahlem et al., 2011). We observed a similar weight loss (Table 2), with rats that received HE3286 losing ~6–10% of their body weight over the course of the study. We observed no adverse effects to general health (e.g., piloerection, hunched posture, decreased responsiveness, labored breathing, sudden lethargy, changes in muscle tone) other than weight loss. We did, however, note thinning of the retina and nuclear layers in rats that received HE3286 (Figure 10). Retinal thickness in vehicle treated rats (~130 μm) was similar to what has been previously reported (Cavallotti et al., 2001; Jiao et al., 2013). Regardless of microbead treatment, retinas from rats treated with HE3286 were 12–34% thinner than rats that received vehicle. This could be a side effect of sterol treatment, as Razali et. al. reported reduced retinal thickness in a model of steroid induced ocular hypertension in rats (Razali et al., 2015). However, HE3286 is not a glucocorticoid or glucocorticoid mimetic, nor does it bind the glucocorticoid nuclear hormone receptor (Ahlem et al., 2011), so this explanation is unlikely. Also, HE3286 treatment did not elevate IOP (Figure 1), which other steroids can do (Razeghinejad and Katz, 2012).

Loss of retinal neurons can lead to thinning of the retina and has been observed in many neurodegenerative disorders or their models, including glaucoma, Alzheimer's disease, multiple sclerosis, and Parkinson's disease (Lee et al., 2014; Vidal-Sanz et al., 2015; Gupta et al., 2016; Knier et al., 2016a). The retinal thinning seen following HE3286 treatment is unlikely to be due to RGC loss, as counts of CTB+/DAPI+ cells (RGCs) in the ganglion cell layer showed no significant differences between vehicle- and HE3286-treated retinas. This was not surprising as RGC soma loss occurs late in the progression of glaucoma (Buckingham et al., 2008; Calkins, 2012). We did see significant thinning of the INL and ONL with HE3286 treatment. This could be a sign of effective treatment. Interestingly, thickening of the INL has been noted in multiple sclerosis patients and correlates with inflammatory disease severity and disease progression (Saidha et al., 2015; Knier et al., 2016a). This increase in INL volume is thought to be due to retinal inflammation; reducing inflammation via immunomodulatory therapy results in thinning of the INL (Saidha et al., 2015; Knier et al., 2016a,b). HE3286 treatment decreased levels of inflammatory markers in this study (Figures 4–6, Table 3). Thinning of the INL and ONL by HE3286 (Figure 10) may reflect a lower inflammatory response in these tissues.

In addition to preserving anterograde axonal transport (Figure 2), HE3286 also mitigated other indicators of dysfunction. For example, treatment with HE3286 resulted in a lack of BDNF elevation in the SC (Figures 3A,D). In a previous study, we observed focal increases in BDNF in regions of the SC where anterograde transport was depleted due to elevated IOP (Crish et al., 2013). Upregulation of BDNF in glaucoma is likely to result from retinorecipient targets responding to glaucoma-relevant stress to improve RGC survival (Chen and Weber, 2004; Lebrun-Julien and Di Polo, 2008; Weber et al., 2010). HE3286's preservation of anterograde transport prevented the need to increase BDNF in the SC, implying that restoration of RGC function (i.e., axonal transport) arrests the complex downstream cascades of gliosis and neuroinflammation normally observed in this condition. Conversely, HE3286 treatment also increased BDNF in the ONH and retina (Figures 3B–D). Neurotrophic factor deprivation due to impaired neurotrophin transport from the brain to the retina is a longstanding hypothesis in the pathogenesis of RGC degeneration in glaucoma (Johnson et al., 2011). Increased BDNF in the optic nerve and retina with IOP elevations suggests that HE3286 acts on the neurotrophin system, either directly, or indirectly by increasing transport, to preserve RGCs (Crish et al., 2013). Whether HE3296 increases BDNF by affecting the trafficking of BDNF or by stimulating local production of BDNF in the retina and optic nerve remains to be determined (Johnson et al., 2011; Crish and Calkins, 2015).

Treatment with HE3286 modulated other proteins implicated in neuroinflammation or neurodegeneration within the optic projection (Figures 5, 6, Table 3). For certain proteins, one dose of HE3286 appeared more effective than the other; this has been observed previously for HE3286 (Ahlem et al., 2009). HE3286 may be more effective at regulating signaling pathways or maintaining homeostasis in a specific tissue at one dose versa another. Microglia become activated in response to injury and upregulate a variety of proteins, including Iba1 (Kreutzberg, 1996; Ito et al., 1998; Soto and Howell, 2014; Mac Nair and Nickells, 2015). Levels of Iba1 were elevated in the SC, ONH, and retina of vehicle-treated rats; this is not surprising since microglial activation has been observed in the optic projection in glaucoma (Wang et al., 2000; Yuan and Neufeld, 2001; Imamura et al., 2009; Ebneter et al., 2010; Shimazawa et al., 2012). HE3286 reduced Iba1 expression in all tissues examined, suggesting it abated microglia activation; a similar effect on microglia was observed in a model of optic neuritis (Khan et al., 2014). Treatment with HE3286 also reduced IL6 levels in the retina and levels of IL6Rαm and TNFα in ONH. Activated microglia produce these pro-inflammatory proteins during glaucoma pathogenesis (Tezel et al., 2001; Sappington and Calkins, 2008; Roh et al., 2012; Sims et al., 2012; Cueva Vargas et al., 2015; Wilson et al., 2015), so it's not unexpected that HE3286 would curb their expression in our model. Interestingly, HE3286 increased levels of IL6 within the SC and IL1β in the retina. While associated with neuroinflammation and implicated in neurodegeneration, both IL6 and IL1β have been shown to be neuroprotective under certain conditions (Sappington et al., 2006; Biber et al., 2008; Chidlow et al., 2012; Matousek et al., 2012; Noguchi et al., 2013; Song et al., 2013; Madeira et al., 2015; Wilson et al., 2015). HE3286 also increased APP levels in saline-injected eyes (Figure 5D). HE3286 is a synthetic derivative of a DHEA metabolite (Ahlem et al., 2011). DHEA levels have been linked to Alzheimer's disease, but the exact role of this neurosteroid in disease progression is currently under debate (Kim et al., 2003; Naylor et al., 2008; Aldred and Mecocci, 2010; Hampl and Bicikova, 2010; Rasmuson et al., 2011). DHEA treatment was neuroprotective in a rat model of AD, and *in vitro* DHEA increases the expression of APP and its cleavage to non-amyloidogenic fragments (Danenberg et al., 1996; Aly et al., 2011). It is possible the increase in APP levels observed in HE3286-treated saline-injected eyes is the result of DHEA-like APP processing. APP can be neuroprotective in some cases (Yankner et al., 1989; Schubert and Behl, 1993; Mucke et al., 1994; Allinquant et al., 1995; Zheng and Koo, 2011). Future studies could include elucidating the effects of HE3286 on APP and its processing in glaucoma and other neurodegenerative diseases. Finally, HE3286 decreased APP and p75 in the ONH and retina, and dramatically reduced ceruloplasmin and C1q expression in the retina of microbead-injected eyes. Each of these factors has been implicated in RGC death, or has been shown to be elevated in the retina and optic nerve in human glaucomatous tissue and/or in models of glaucoma (Stasi et al., 2006, 2007; Goldblum et al., 2007; Stevens et al., 2007; Wei et al., 2007; Lebrun-Julien et al., 2009; Kipfer-Kauer et al., 2010; Howell et al., 2011, 2014; Ding et al., 2012; Meeker and Williams, 2015). Taken as a whole, it appears HE3286 mitigates the neuroinflammatory response by dampening microglial activation.

The expression of TNFα, IL6, APP in neurons and glia, as well as the production of β-amyloid, and the activation of microglia can all be regulated by NFκB activation (Mattson and Camandola, 2001; Camandola and Mattson, 2007). A suggested mechanism of action for HE3286 is the regulation of NFκB activity (Auci et al., 2007; Ahlem et al., 2009, 2011; Offner et al., 2009). NFκB is a transcription factor activated by a wide range of stimuli that in turn regulates the expression of genes involved inflammation, immune response, cell survival and cell death (Gilmore, 2008; Hayden and Ghosh, 2008; Lawrence, 2009). Activated NFκB translocates to the nucleus, and we observed nuclear localization of NFκB in neurons and glia within the optic projection of vehicle- and HE3286-treated rats following IOP elevation (Figures 7–9). Similar activation of NFκB in the visual pathway has been observed following injury and in models of glaucoma (Choi et al., 1998; Agapova et al., 2006; Haenold et al., 2014). Treatment with HE3286 increased NFκB localization in neuronal nuclei within the SC and retina and decreased NFκB localization in ONH glial nuclei in microbead-injected eyes. This cell-specific NFκB activation may explain the beneficial outcomes observed in HE3286-treated rats; NFκB activation in glial cells promotes neuronal degeneration, while activation in neurons promotes survival (Mattson and Camandola, 2001; Camandola and Mattson, 2007). Activated NFκB can enhance mitochondrial bioenergetics and prevent peripheral neuropathy in rodent models of diabetes (Saleh et al., 2013). Increased NFκB localization to RGC nuclei in HE3286-treated microbead-injected eyes may protect these neurons metabolically (Kong et al., 2009; Baltan et al., 2010; Lee et al., 2011; Coughlin et al., 2015; Kleesattel et al., 2015; Fahy et al., 2016), such that axonal transport is preserved compared to vehicle-treated eyes (Figure 2). Whether or not HE3286 maintains RGC functionality could be assessed using outcomes such as visual evoked potentials, electroretinogram, or two-alternative forced-choice visual behavioral testing in this or other models of glaucoma.

HE3286 has produced therapeutic benefits in various models of inflammatory disease, in a model of Parkinson's and in clinical trials for diabetic complications (Auci et al., 2007, 2010; Ahlem et al., 2009; Offner et al., 2009; Conrad et al., 2010; Lu et al., 2010; Kosiewicz et al., 2011; Nicoletti et al., 2012; Reading et al., 2013a,b; Khan et al., 2014). HE3286 has been well tolerated and has presented no adverse effects in human trials (Reading et al., 2013a,b). We observed positive therapeutic outcomes in terms of anterograde axonal transport, the expression of proteins associated with neuroinflammation, neurodegeneration, and glaucoma pathogenesis, and NFκB activation. The results of this study suggest HE3286 provides neuroprotection to RGCs and their axons, which helps preserve function in these neurons following exposure to elevated ocular pressure. This neuroprotection could be due to caloric restriction (Table 2), as has been observed in a mouse model of glaucoma (Guo et al., 2016). However, HE3286's anti-inflammatory activity has been observed *in vitro* and in mice without weight loss compared to vehicle controls, and in humans without caloric restriction or weight loss (Wang et al., 2010; Reading et al., 2013b). A matched feeding experimental design would be needed to understand the potential contribution of reduced caloric intake in our glaucoma model, but prior evidence in other models suggest the current results are not predominately a result of caloric restriction. Glaucoma shares many common features with other neurodegenerative diseases (Crish and Calkins, 2011; Mckinnon, 2012; Ghiso et al., 2013; Jindal, 2013; Danesh-Meyer and Levin, 2015; Jain and Aref, 2015). Given the number of people currently with or likely to develop glaucoma in the near future, the commonalities between glaucoma and other age-related neurodegenerative diseases, and that HE3286 is already being tested in clinical trials, further studies on HE3286 and the mechanism of its action in the central nervous system is warranted.

## Author contributions

WL contributed substantially to the experimental design, the acquisition, analysis and interpretation of data, and the writing and revising the manuscript. BC, CF contributed substantially to the acquisition and analysis of data and assisted in manuscript revisions. RS contributed substantially to the experimental design with regards to neuroinflammation, and assisted in the critical revision of the manuscript. CA contributed substantially to the conception and experimental design, and assisted in the critical revision of the manuscript. DC contributed substantially to the conception and experimental design, interpretation of data, and assisted in the critical revision of the manuscript.

## Funding

The authors declare that this research was supported by a grant from Harbor Therapeutics, Inc. (San Diego, CA). We also acknowledge support from the Vanderbilt Vision Research Center (5P30EY008126, DC).

### Conflict of interest statement

The authors declare that this research was supported by a grant from Harbor Therapeutics, Inc (San Diego, CA). The reviewer MH and handling Editor declared their shared affiliation, and the handling Editor states that the process nevertheless met the standards of a fair and objective review.
